# Impact of SARS-CoV-2 Infection on Maternal and Neonatal Outcome in Correlation with Sociodemographic Aspects: A Retrospective Case-Control Study

**DOI:** 10.3390/jcm12196322

**Published:** 2023-09-30

**Authors:** Radu Chicea, Andrei Dorin Neagu, Eugen Dan Chicea, Amina Simona Grindeanu, Dan Georgian Bratu, Adrian Gheorghe Boicean, Mihai Dan Roman, Sorin Radu Fleacă, Liana Maria Chicea, Dumitru Alin Teacoe, Ioana Andrada Radu, Maria Livia Ognean

**Affiliations:** 1 Faculty of Medicine, Lucian Blaga University Sibiu, 550024 Sibiu, Romania; radu.chicea@ulbsibiu.ro (R.C.); livia_sibiu@yahoo.com (M.L.O.); 2 Emergency Clinical County Hospital Sibiu, 550245 Sibiu, Romania

**Keywords:** SARS-CoV-2 infection, pregnancy, maternal outcome, birth, Delta, Omicron

## Abstract

Background: As the COVID-19 pandemic evolved, concerns grew about its impact on pregnant women. This study aimed to determine how SARS-CoV-2 affects pregnancy, birth, and newborns, in order to identify vulnerable individuals and provide proper care. Methods: This is a retrospective case-control study of 398 pregnant women who delivered at the Emergency Clinical County Hospital in Sibiu, Romania from 1 February 2020 to 31 March 2022. Patients were initially grouped and compared based on their RT-PCR SARS-CoV-2 test results into the COVID group (cases) (N = 199) and non-COVID group (control) (N = 199). The COVID cases were further divided and compared according to the pre-Delta (N = 105) and Delta/Omicron (N = 94) SARS-CoV-2 variants. COVID cases and control groups were compared to identify correlations between sociodemographic factors, pregnancy outcomes, and SARS-CoV-2 infection. The same comparisons were performed between pre-Delta and Delta/Omicron groups. Results: There were no significant differences concerning maternal residence, while educational level and employment proportion were higher among the positively tested patients. No significant differences were found for neonatal and pregnancy complications between COVID cases and control groups. Except for a lower mean gestational age, no significant differences were found between pre-Delta and Delta/Omicron periods. The maternal mortality in the infected group was 0.5% (1 case). Conclusions: Our study showed that SARS-CoV-2 infection at birth did not significantly affect maternal and neonatal outcomes, not even considering the SARS-CoV-2 strain.

## 1. Introduction

COVID-19 (Coronavirus disease 2019) is an infectious disease caused by the SARS-CoV-2 virus, a single-stranded RNA virus. The first reported case of a highly contagious coronavirus strain, known as the severe acute respiratory syndrome (SARS-CoV) was in China in 2002. Meanwhile, its genetic material has been subjected to various changes, some of which have altered the transmission and severity of the disease, leading eventually to the occurrence of the novel SARS-CoV-2 variant, which originated in Wuhan, China towards the end of 2019 [1,2,3]. 

Since its emergence, SARS-CoV-2 has had a devastating impact on global demographics, resulting in over 6,347,816 deaths worldwide as of July 2022. This has made it the most significant global health crisis in recent times. It quickly spread across North and South America, Europe and Asia, resulting globally in over 552,504,629 confirmed cases, of which 2,927,187 have been registered in Romania [4,5]. Transmission occurs by contact with infected wastes, secretions and droplets. The clinical course of the disease appears to follow a triphasic pattern and initially has an incubation period of 2 to 16 days [1,2,3,6]. Fever, myalgia, headache and diarrhea are prodromal symptoms dominating the first week of infection. Patients may develop symptoms such as recurrent fever, watery diarrhea, a dry and unproductive cough, and mild difficulty breathing during the second week, all of these occurring at the same time with the falling of viral load and IgG seroconversion. The overactive immune response of the host is believed to be the reason for disease progression at this stage. Progressing to SARS is the third and occasionally fatal phase, affecting 20% of patients [7,8,9].

Most individuals infected with the virus experience a range of respiratory symptoms from mild to severe, and can recover without needing hospitalization. However, some may develop severe illness that requires specialized medical care. Individuals who are older and those with underlying medical conditions such as cancer, diabetes, cardiovascular diseases, or chronic respiratory diseases are at a higher risk of developing severe illness [10,11,12,13]. Pregnant women appear to be at high risk of severe infection, which can lead to maternal, obstetrical and neonatal complications. Both sociodemographic factors (maternal age, household, education level and income), as well as pregnancy related aspects (gestational age, parity, pathology, placental anomalies and delivery mode) can significantly affect maternal, perinatal and neonatal outcomes [14,15,16,17,18,19,20,21,22].

Transmissibility, pathogenicity and public health measures have experienced significant alterations following the emergence of SARS-CoV-2 variants of concern. Our study compared both the impact of the SARS-CoV-2 variant wave on hospitalized patients, as well as the differences between the infected group and the non-infected control group of mothers and newborns, using sociodemographic, clinical and paraclinical data. The progression of the COVID-19 pandemic has raised significant concerns regarding the prevention and treatment of COVID-19 during pregnancy, as well as the potential risks of SARS-CoV-2 infection for both the mother and the newborn [20,21,23,24,25,26,27,28,29,30,31,32,33]. This study sought to assess the effects of SARS-CoV-2 infection at birth on maternal and neonatal health outcomes, including morbidity and mortality. Identifying the at-risk population is crucial for providing optimal care.

## 2. Materials and Methods

This retrospective case-control study involved pregnant women who have been admitted with confirmed SARS-CoV-2 infection at the Emergency Clinical County Hospital of Sibiu, Romania from 1 February 2020 to 31 March 2022. During the study period, all pregnant women at the hospital for delivery were screened according to the hospital protocol, for the presence of symptoms of SARS-CoV-2 infection (fever, cough, dyspnea, odynophagia, myalgia, tiredness, loss of taste or smell, headache, difficulty in breathing or shortness of breath, chest pain), epidemiological report of travel or/and direct exposure history. Based on the results of the RT-PCR (real-time polymerase chain reaction) screening obtained through oropharyngeal and nasopharyngeal swabs, all pregnant women who delivered at the Sibiu Emergency Clinical County Hospital during the study period were separated into a COVID group and a non-COVID control group. Newborns delivered by SARS-CoV-2 mothers were also tested using RT-PCR at 24 and 48 h after delivery and re-tested at 72 h if one of the previous tests was positive or inconclusive. The control group consisted of matched 1:1 pregnant women of the same age, delivering at the same gestational age, within a 2–3 weeks period around the delivery date of the case patient. A total of 398 pregnant women and their newborns were included in the case-control study: 199 pairs with confirmed maternal SARS-CoV-2 infection (cases) and 199 without maternal SARS-CoV-2 infection (control group). Furthermore, all confirmed cases were divided into pre-Delta (before June 2021) and Delta/Omicron (starting 1st June 2021) groups, based on data on strain dominance as reported by the National Institute of Public Health Data. The patients were observed and monitored until delivery and pregnancy, maternal and neonatal outcome data were collected, together with maternal sociodemographic aspects. The primary outcomes were pregnancy-related (maternal death, gestational age at delivery, pregnancy complications, and delivery mode). The study’s secondary outcomes included the rates of preterm delivery, neonatal birth weight, and 1-min Apgar scores. The study database included data collected from maternal and neonatal medical records, such as: maternal age, maternal residence (urban/rural), body mass index (BMI), smoking history, educational level, gestational age, gravidity and parity, Apgar score (1 min), neonatal RT-PCR test results, delivery mode, indication of Caesarean Section, placental abnormalities, pregnancy associated pathologies, clinical features and mortality of SARS-CoV-2. Placentas were macroscopically analysed for abnormalities in all cases. No microscopical examinations of placentas were performed. 

The IBM SPSS 23 was used for data analysis. Descriptive statistics were utilized to determine the normal distribution of numerical data. Mean values and standard deviation were used to present the results of independent samples *t*-tests conducted on numerical variables with normal distribution. Median values and interquartile (25th–75th) range were calculated, presented, and compared using Mann-Whitney test for variables with abnormal distribution. Fisher’s Exact Test or Pearson chi-square tests were used for analysis of categorical variables. OR with a 95% confidence interval (CI) were calculated when appropriated, a *p* < 0.05 was chosen as cut-off for statistical significance.

For a better understanding, we ought to define some terms further used, such as: gestational hypertension as elevated blood pressure higher than 140/90 mmHg, occurring for the first time in the second half of the pregnancy, without proteinuria. Preeclampsia was defined as gestational hypertension with elevated blood pressure above 140/90 mmHg, and proteinuria. Pregnant women aged over 35 years were considered elderly. Fetal distress was diagnosed during labor whenever the fetus presented signs of improper oxygenation (changes of fetal heart rhythm, abnormal cardiotocogram, abnormal amniotic fluid color and consistency). Prematurity was defined as birth occurring before 37 completed gestational weeks. Macrosomia was defined as birth weight above 4000 g. 

## 3. Results

In our clinic, there were 6666 births during the study period, so COVID-19 incidence was 2.98%. The incidence of pre-Delta cases was 2.54%, while for the Delta/Omicron cases it was 3.68%. Prematurity occurred in 485 patients during the study period, out of which 9 were from COVID-19 infected, with an incidence of 1.85%. A total of 4 cases were in the pre-Delta period, with an incidence of 1.39%, while 5 were in the Delta/Omicron period with an incidence of 2.51%.

### 3.1. SARS-CoV-2 Infection Group vs. CONTROL Group

Out of a total of 199 SARS-CoV-2 confirmed maternal infections (cases), 69 cases (34.7%) were admitted in 2020, 73 (36.7%) in 2021 and 57 (28.6%) in 2022. 184 (92.5%) of the cases were asymptomatic and 15 (7.5%) were symptomatic. Out of these, 14 developed mild to moderate forms of illness, while just 1 (0.5%) patient needed mechanical ventilation.

The mean (standard deviation—SD) maternal age of the SARS-CoV-2 infection group was 27.03 (6.13), 14.7% of the patients having under 18 years of age. 50.7% patients were living in urban areas. Most of the patients were highly educated (80/40.2%), followed by those with middle educational level (60/30.1%), those graduating primary school (39/19.6%), while 20 (10.1%) patients were illiterate. Out of the 199 infected pregnant women, 132 (66.3%) were employed. 

As BMI was described in the literature as a risk factor for SARS-CoV-2 infection, we analysed BMI in correlation with SARS-CoV-2 infection around delivery: 28 (14.1%) patients had a normal weight, 106 (53.3%) were overweight, 50 (25.1%) had class 1 obesity, 8 (4%) had class 2 obesity and 7 (3.5%) were classified with class 3 obesity, the mean (SD) BMI in the study group being 28.16 ± 4.72. The control group had a mean (SD) BMI of 29.06 ± 4.20, significantly higher compared to the SARS-CoV-2 group (*p* = 0.045). The percentage of patients classified as obese was higher in the control group (38.7%) as compared to the SARS-CoV-2 infected group, where only 32.7% of patients were found to be obese. Between the 2 groups, no differences were identified as regards to smoking history, both groups having a total number of 45 (22.6%) smokers. 

Regarding the follow-up protocol of the pregnancy, 11 pregnant women (5.5%) were incorrectly followed-up in the SARS-CoV-2 infection group, compared to 27 (13.6%) in the control group. 

The mean (SD) gravidity of the SARS-CoV-2 group was 1.72 ± 0.99, while the mean (SD) parity was 1.99 ± 1.23.

Most of the pregnant women delivered at 39 weeks gestational age (GA) (124 cases/31.1%), followed by delivery at 40 weeks GA (112/28.1%) and 38 weeks GA (100/25.1%). A total of 50 patients (12.5%) delivered prematurely, the lowest GA being 26 weeks (1 case). The mean (SD) GA in both groups was 38.67 ± 1.740 weeks, as it was a matched criteria for selecting the control group cases.

There was no significant difference between delivery mode: in the SARS-CoV-2 group, 107 patients had vaginal delivery (53.8%) compared to 119 (59.8%) patients in the control group (*p* = 0.133). History of previous C-Section was the main indication for C-Section delivery, described in 28 SARS-CoV-2 group cases (30.4%) vs. 15 in the control group (18.8%), followed by fetal distress (7/7.6% vs. 9/11.3%), gestational hypertension (4/4.3% vs. 6/7.5%), thrombophilia (5/5.4% vs. 4/5%), lack of labor progression (5/5.4% vs. 13/16.3%), fetal macrosomia (4/4.3% vs. 2/2.5%) and elderly primipara (3/3.3% vs. 3/3.8%) (Table 1).

Placental abnormalities (placenta praevia and low-lying placenta) were observed in a total of 10 cases, of which 6 in the SARS-CoV-2 group (5 cases with low-lying placenta; 1 with placenta praevia) and 4 in the control group (3 cases with low-lying placenta; 1 case of placenta praevia) (*p* = 0.667) (Table 1). Placentas were macroscopically analysed for abnormalities in all cases, but none were found. No microscopic examinations of placentas were performed. In other specialized studies it was shown that pregnant women appeared to have an increased risk for pregnancy complications. Pregnancy-associated pathologies were documented in a total number of 93 cases, 52 (26.1%) in SARS-CoV-2 positive patients and 41 (20.6%) among the control group. The most frequent pathology involved the genitourinary tract, 20 patients (5%) having a history of urinary tract infections (UTI) (19/9.5% in SARS-CoV-2 group vs. 1/0.5% in control group). Other frequent pregnancy complications were gestational hypertension 17 (4.3%) (5/2.5% vs. 12/6%), gestational diabetes 8 (2%) (4/2% vs. 4/2%), and severe gestational anemia 12 (3%) (2/1% vs. 10/5%). Of the 6666 deliveries during the study period, 52 cases of preeclampsia were diagnosed, with an incidence of 0.78%. Only 1 case (0.5%) of preeclampsia was diagnosed in our study, occurring during the Delta/Omicron period. Only 1 maternal death was noted, in the SARS-CoV-2 infection group (0.5% incidence), which, ultimately, was caused by myocardial infarction.

The neonates delivered by SARS-CoV-2 infected mothers were tested using RT-PCR at 24 h and 48 h after delivery, 3 newborns were tested positive at 24 h, 2 negative at 48 h, and 1 consistently positive at 48 h and 72 h. Other 2 newborns were negative at the first test, but positive at 48 h and 72 h, while1 newborn had an inconclusive test at 24 h and 2 negative tests at 48 h and 72 h. Finally, 1 newborn had a negative test at 24 h and 72 h but an inconclusive test at 48 h. In summary, 3 newborns were SARS-CoV-2 infected, 2 of which during the Delta/Omicron period, and all of them were asymptomatic.

### 3.2. Pre-Delta Group vs. Delta/Omicron Group

**Figure 1:**
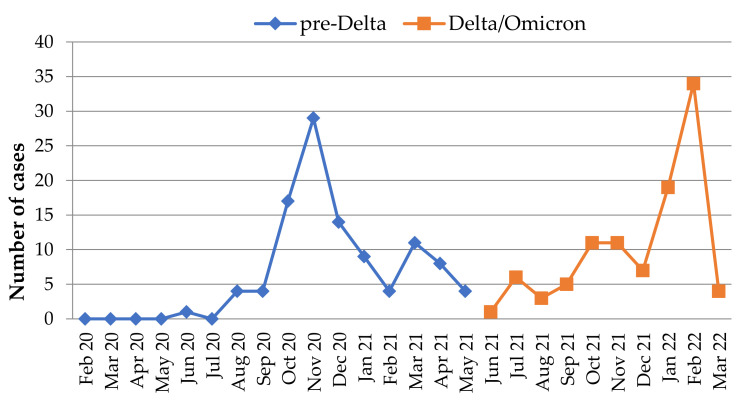
Monthly distribution of maternal SARS-CoV-2 infections (pre-Delta-Delta/Omicron).

The analysis was extended to evaluate the impact of SARS-CoV-2 infection during delivery on pregnancy, maternal and neonatal morbidity and mortality, after dividing the SARS-CoV-2 infection cases into pre-Delta (before June 2021) (N = 105/52.8%) and Delta/Omicron groups (starting June 2021) (N = 94/47.2%) (Figure 1). As no genotyping was possible, we divided our cases according to the National Institute of Public Health Data. 

The mean (SD) maternal age of patients who tested positive for RT-PCR SARS-CoV-2 test was 27.41 ± 6.103 in the pre-Delta period, whereas in the Delta period the mean (SD) maternal age was 26.64 ± 6.152, *p* = 0.378 (statistically not significant). Most of the pregnant women had urban residence, in pre-Delta period (58/55.2%) while most patients had rural residence in the Delta/Omicron period (51/54.3%). Fewer smokers were documented among patients in the pre-Delta period (19/18.1%) vs. Delta/Omicron period (26/27.6%) but the difference was not statistically significant (*p* = 0.081). There were no differences identified between the groups regarding obesity prevalence (defined as BMI > 30): 39 obese patients (37.1%) in the pre-Delta Group vs. 26 (27.6%) in the Delta/Omicron group (*p* = 0.093). Also, no difference was noted considering the pregnancy follow-up protocol, 11 (5.5%) patients were incorrectly followed-up—6 (5.7%) in the pre-Delta period vs. 5 (5.3%) during Delta/Omicron period.

The mean (SD) GA for pre-Delta SARS-CoV-2 group was 38.95 ± 1.28 weeks, significantly higher compared to the mean GA of the Delta/Omicron group (38.35 ± 2.09), *p* = 0.014, but without clinical significance as most of the newborns were delivered around 38 weeks. No difference was found between pre-Delta and Delta/Omicron groups concerning mean (SD) gravidity and parity (*p* = 0.736, and *p* = 0.350 respectively) (Table 2).

The rates of pregnancy-associated conditions were 52 (26.1%), with 21 (20%) in the pre-Delta group vs. 31 (33%) in the Delta/Omicron group. Comparing the pre-Delta to Delta/Omicron group, the most common comorbidities observed were UTI—8 (7.6%) vs. 11 (11.7%), gestational hypertension—2 (1.9%) vs. 3 (3.2%), gestational diabetes—1 (1%) vs. 3 (3.2%)—and colpitis—8 (7.7%) vs. 3 (3.2%). 

Analysis of placental abnormalities during the 2 study periods showed no difference—6 (3%) patients had placental abnormalities, 2 (1.9%) in the pre-Delta period and 4 (4.2%) during Delta/Omicron period, *p* = 0.295 (Table 3).

The majority of the patients delivered vaginally: 107 (53.8%) − 50 (47.6%) in pre-Delta group vs. 57 (60%) in Delta/Omicron group. The most frequent indication of C-Section delivery was history of previous C-Section, described in 28 (30.1%) of the cases (16/29.1% in the pre-Delta group vs. 12/32.4% in the Delta/Omicron group), followed by SARS-CoV-2 infection (considered as indication per protocol at a certain time during COVID-19 pandemic). Other less common indications were: acute fetal distress, gestational hypertension/preeclampsia, thrombophilia, and gestational diabetes (Table 3). 

The relationship between maternal SARS-CoV-2 infection and neonatal outcomes was evaluated using 1 min Apgar score and neonatal birth weight. No significant differences were found between the study groups, as shown in Table 2.

Focusing on data about the clinical evolution of SARS-CoV-2 infection during pre-Delta vs. Delta/Omicron period, we found that out of the total of 199 RT-PCR positive pregnant women, just 15 (7.5%) were symptomatic around delivery, significantly more during Delta/Omicron wave (4/3.8% in the pre-Delta group vs. 11/11.7% in the Delta/Omicron group), *p* = 0.034. Most of the patients developed a mild illness (symptoms of COVID-19 without shortness of breath), 11 (5.5%) (with 3/2.9% in the pre-Delta period vs. 8/8.5% during Delta/Omicron period), while 3 (1.5%) (with 1/0.9% vs. 2/2.1%) had moderate illness (defined as the need of oxygen therapy) and just 1 patient (0.5%) from the Delta/Omicron group needed mechanical ventilation (Table 3).

## 4. Discussion

The present study included 199 women with SARS-CoV-2 infection, diagnosed around delivery, during the second and third trimester of pregnancy, and 199 control pregnant women, matched by gestational and maternal age, who delivered in our clinic during the study period, from 1 February 2020 to 31 March 2022. 

The mean maternal age in the studied population was 27.03 years, while the mean gestational age was 39 weeks. The rate of preterm delivery in our study was considerably lower (12.5%) than the rate identified by Villar et al [33] and Jering et al [34], studies that reported that maternal SARS-CoV-2 infection was associated with higher odds of preterm birth. Our results are consistent with those reported by Yalçin et al from Turkey, who found no difference in preterm birth rate between women with and those without SARS-CoV-2 infection [35].

We analysed possible correlations between some sociodemographic aspects (educational level, maternal residence and employment) and SARS-CoV-2 infection and found no differences regarding the residence, while educational level and employment were higher, but not significantly, among the positive tested patients for SARS-CoV-2 infection. Strongly related to these sociodemographic aspects, we found out that almost all infected patients were correctly followed-up during pregnancy. Patients with higher education may have had a better understanding of COVID-19 implications for their pregnancy and better acknowledgment of SARS-CoV-2 clinical manifestations and importance of an adequate follow-up during pregnancy, leading to an increased accessibility to medical services. Also, these patients are prone to get in contact with more people due to work-related demands. 

Our findings do not concur or compete with those of systematic literature reviews which state that women who actively smoke are less likely to be represented among those with COVID-19, as we found, surprisingly, the same number of active smokers in both groups, with and without SARS-CoV-2 infection. But as there were contradictory opinions in the specialized literature, it remains to be further investigated if smoking is a real risk factor for SARS-CoV-2 infection or if it has a protective role [36].

Obese people have a higher risk of contracting COVID-19, probably due to hormone and nutrient dysregulation, which can affect the body’s ability to respond to infection. In our study group of pregnant patients, obesity did not increase the risk of SARS-CoV-2 infection [37]. Again, contrary to data in the literature, more obese pregnant women were found in the control group [38]. Also, there were no significant differences found between the pre-Delta and Delta/Omicron groups, with only 13 more cases of obese patients in the pre-Delta group.

Our results are similar to previous literature articles regarding delivery mode, as we noted only 6% more C-Section deliveries among the SARS-CoV-2 infected group compared to the control one [39]. Our explanation is that, at the beginning of the pandemic, the first official recommendation and protocol stated that SARS-CoV-2 infected confirmed cases should undergo delivery via C-Section, as it was thought that it may reduce the risk of transmission to the medical personnel compared to the vaginal birth, especially during the second stage of labor, when the expulsive efforts increase the amount of droplets in the air and ultimately raise the risk of viral transmission. In addition, in order to save personal protective equipment, due to restricted availability, the majority of hospitals decided to employ C-Sections as the primary delivery method. Our results concur with those previously mentioned, as we noted a higher proportion of pregnancies terminated through C-Section during the pre-Delta period, when SARS-CoV-2 infection was a firm indication for surgical delivery. Considering the outcomes for both the mother and neonate in SARS-CoV-2 infected cases, there is still a lack of conclusive information as to which delivery method is preferable, therefore, the current protocol states that the delivery method should be individualized based on medical and obstetrical indications [40]. Other indications for C-Section delivery, besides SARS-CoV-2 infection, were, in order of prevalence, history of previous C-Section, fetal distress, gestational hypertension and thrombophilia, relatively similar for both SARS-CoV-2 infected and non-infected pregnant women. 

A recent meta-analysis showed that maternal complications in SARS-CoV-2 infected pregnant women groups tend to be higher than in the general population [14]. The most frequent comorbidities documented in our study were UTIs and gestational hypertension, followed by severe gestational anemia and gestational diabetes. Despite the general consensus that SARS-CoV-2 infection would increase the risk of gestational hypertension which is related to endothelial dysfunction and coagulation disorders, the prevalence of this obstetrical complication, in our study, was only 3.5% higher in the control group compared to the infected group. Also, no notable variations of gestational hypertension existed between our pre-Delta and Delta/Omicron groups. Upon assessing the recorded placental abnormalities, our analysis revealed no statistically significant differences between the groups under investigation. No macroscopic placental abnormalities were discovered, therefore no anatomopathological examinations were recommended, according to our unit protocol. It is highly likely that the limited number of cases contributed to this outcome.

According to medical literature, there is a significantly higher risk of adverse neonatal outcomes, need for specialized neonatal intensive care and/or prolonged neonatal admission after birth for newborns born from SARS-CoV-2 infected mothers [20,22]. Most probably due to the fact that our study group mean GA was 39 weeks, we found no notable differences in neonatal adverse outcomes, the mean 1 min Apgar score being over 9. All neonatal intensive care unit (NICU) admissions were due to common reasons, none because of neonatal SARS-CoV-2 infection. No association was found between fetal distress and maternal SARS-CoV-2 infection. 

Our results concerning COVID-19 clinical manifestations reached statistical significance and were in line with previous literature data; the majority of the SARS-CoV-2 maternal infections were asymptomatic on admission and, out of the symptomatic ones, most of them developed a mild illness. Only one patient needed mechanical ventilation after giving birth and resulted in the single maternal death in our study, again, contradicting the current reports demonstrating a significantly increased risk of maternal death in SARS-CoV-2 positive cases [39,40].

Our study has a number of limitations, such as, firstly, having a relatively small size of the study group, possibly leading to a lack of statistical significance in some comparisons. It is essential to interpret our findings as applicable solely to the outcome of a SARS-CoV-2 test conducted at the time of delivery, rather than related to infection occurring at any time during pregnancy. Another limitation was the fact that we covered in our study only pregnant women with a positive RT-PCR SARS-CoV-2 test at the time of birth. As COVID-19 may surge again, the results of our study may be useful in evaluating pregnant women admitted for delivery. More information may be collected in order to find significant correlations between maternal COVID-19 at birth and maternal and neonatal outcomes.

## 5. Conclusions

Regardless of the sociodemographic aspects and the COVID-19 pandemic period, our study showed that SARS-CoV-2 infection irrespective of the virus variant, at birth, did not significantly affect the maternal and neonatal outcomes. Further research is needed to establish the impact of contracting SARS-CoV-2 infection during earlier stages of pregnancy on the maternal, fetal and neonatal outcomes. Both the optimal type of delivery and the vertical transmission also remain subjects of interest for future studies.

## Figures and Tables

**Table 1:** Comparison of pregnancy and labor related data between SARS-CoV-2 and control groups.

Variables		Cases (Total = 199)	Control (Total = 199)	Odds Ratio (95% CI)	*p*
Delivery mode	Vaginal (*n*/%)	107 (53.8%)	119 (59.8%)	0.78 (0.52–1.16)	0.133
	C-Section (*n*/%)	92 (46.2%)	80 (40.2%)		
C-Section indication	History of previous C-Section (*n*/%)	28 (30.4%)	15 (18.8%)	-	-
	Fetal distress (*n*/%)	7 (7.6%)	9 (11.3%)	-	-
	Gestational hypertension (*n*/%)	4 (4.3%)	6 (7.5%)	-	-
	Thrombophilia (*n*/%)	5 (5.4%)	4 (5%)	-	-
	Lack of labor progression(*n*/%)	5 (5.4%)	13 (16.3%)	-	-
	Fetal macrosomia (*n*/%)	4 (4.3%)	2 (2.5%)	-	-
	Elderly primipara (*n*/%)	3 (3.3%)	3 (3.8%)	-	-
Placental abnormalities (*n*/%)		6 (3%)	4 (2%)	1.66 (1.00–2.76)	0.667
Obesity (*n*/%)		65 (32.7%)	77 (38.7%)	1.30 (0.86–1.96)	0.125
Smoking history (*n*/%)		45 (22.6%)	45 (22.6%)	-	-
Pregnancy associated conditions (*n*/%)		52 (26.1%)	42 (21.1%)	-	0.268

**Table 2:** Comparison of various maternal characteristics between pre-Delta and Delta/Omicron groups.

Variables	Pre-Delta (Total = 105)	Delta/Omicron (Total = 94)	*p*
Maternal age (median, IQR 25th–75th) (years)	27 (23–31.5)	27 (22–31.0)	0.362 ^†^
BMI (median, IQR 25th–75th)	28 (24.95–31.25)	27 (24.9–30.1)	0.193 ^†^
Gestational age (mean ± SD) (weeks)	38.95 ± 1.28	38.35 ± 2.09	0.014 *
Gravidity (median, IQR 25th–75th)	2 (1–3)	2 (1–2)	0.463 ^†^
Parity (median, IQR 25th–75th)	2 (1–2)	1 (1–2)	0.778 ^†^
Apgar score 1′ (mean ± SD)	9.60 ± 0.87	9.37 ± 1.11	0.101 *
Birth weight (mean ± SD) (grams)	3212.1 ± 479.71	3103.3 ± 559.49	0.140 *

^†^ Variables with abnormal distribution were compared using Mann Whitney test. * Variables with normal distribution were compared using Independent T Samples test. IQR—interquartile range, BMI—body mass index.

**Table 3:** Comparison of various data between pre-Delta and Delta/Omicron groups.

Variables		Pre-Delta (Total = 105)	Delta/Omicron (Total = 94)	OddsRatio (95% CI)	*p*
Type of delivery	Vaginal (*n*/%)	50 (47.6%)	57 (60%)	0.60 (0.35–1.06)	0.053
	C-Section (*n*/%)	55 (52.4%)	37 (40%)		
Indication of C-Section	COVID (*n*/%)	19 (34.5%)	4 (10.8%)	-	-
	History of previous C-Section (*n*/%)	16 (29.1%)	12 (32.4%)		
	Fetal Distress (*n*/%)	4 (7.3%)	3 (8.1%)		
	Gestational hypertension (*n*/%)	2 (3.6%)	2 (5.4%)		
	Thrombophilia (*n*/%)	4 (7.3%)	1 (2.7%)		
	Gestational Diabetes (*n*/%)	2 (3.6%)	1 (2.7%)		
Placental Abnormalities (*n*/%)		2 (1.9%)	4 (4.2%)	2.26 (0.40–12.65)	0.295
Severity	Asymptomatic (*n*/%)	101 (96.2%)	83 (88.3%)	-	0.037
	Total (*n*/%)	4 (3.8%)	11 (11.7%)	3.31 (1.02–10.76)	0.034
	Mild (*n*/%)	3 (2.9%)	8 (8.5%)	-	-
	Moderate (*n*/%)	1 (0.9%)	2 (2.1%)	-	-
	Mechanical ventilation (*n*/%)	0 (0%)	1 (1.1%)	-	-
Obesity (*n*/%)		39 (37.1%)	26 (27.6%)	0.64 (0.35–1.16)	0.093
Smoking History (*n*/%)		19 (18.1%)	26 (27.6%)	1.71 (0.87–3.33)	0.081
Education	No education (*n*/%)	8 (7.6%)	12 (12.7%)	-	-
	Primary education (*n*/%)	21 (20%)	18 (19.1%)		
	Middle education (*n*/%)	30 (28.6%)	30 (31.9%)		
	High education (*n*/%)	46 (43.8%)	34 (36.2%)		
Place of residence	Urban (*n*/%)	58 (55.2%)	43 (45.7%)	1.49 (0.85–2.61)	0.102
	Rural (*n*/%)	47 (44.8%)	51 (54.3%)		
Pregnancy associated conditions (*n*/%)		21 (20%)	31 (33%)	1.94 (1.02–3.68)	0.031
SARS-CoV-2 diagnostic	Prenatal (*n*/%)	65 (61.9%)	74 (78.7%)	0.46 (0.25–0.86)	0.010
	Postnatal (*n*/%)	40 (38.1%)	20 (21.3%)		

## Data Availability

The data presented in this study are available on request from the corresponding author. The data are not publicly available due to privacy reasons.
